# Screening for atrial fibrillation: less is more?

**DOI:** 10.1093/ehjopen/oeab038

**Published:** 2021-12-31

**Authors:** Emma Svennberg, Frieder Braunschweig

**Affiliations:** Department of Cardiology, Karolinska University Hospital, SE-141 86 Stockholm, Sweden

## Abstract

**Graphical Abstract:**
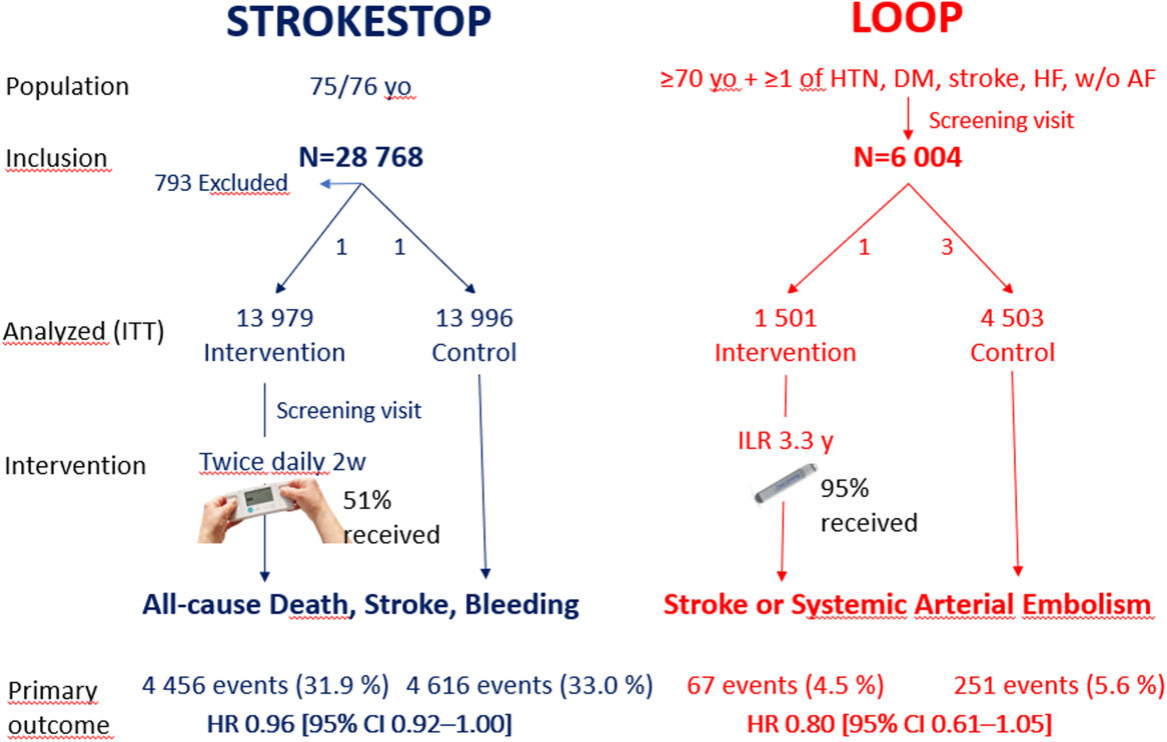

Modern technology offers an ever-increasing range of tools to monitor heart rhythm. An apparent potential of such applications is the detection of unknown atrial fibrillation (AF) in persons with an increased risk of thrombo-embolic stroke. Yet, the impact of such screening on clinical outcomes has been unclear.

Recently, two large trials, STROKESTOP and LOOP,^1^^,^^2^ reported differing findings making it worthwhile to discuss promises and pitfalls of systematic AF screening in risk groups.

STROKESTOP, using a population-screening approach, randomized 28 768 residents in two Swedish regions aged 75/76 to be invited to screening or control. In the screening group, participants without known AF performed a hand-held single-lead ECG twice daily for 2 weeks. Those with known AF but without stroke-protective treatment were offered oral anticoagulant therapy (OAC). Of 13 979 invited for screening, 51% chose to participate.

In LOOP, individuals aged 70–90 with at least one additional risk factor for stroke but without known AF or OAC were identified from registries and invited to attend a screening centre. The 6004 participants were then randomized 1:3 to receive an implantable loop recorder (ILR) or to control. Of those randomized to ILR, 95% received a device.

Both studies were analysed according to ‘intention to screen’ and had no loss of follow-up (median 6.9 years in STROKESTOP; 5.4 years in LOOP). The primary endpoint of STROKESTOP, a composite of stroke, systemic embolism, all-cause mortality, and major bleeding, was significantly lower in the screening group [hazard ratio (HR) 0.96, 95% confidence interval (CI) 0.92–1.00]. In LOOP the primary endpoint was stroke or systemic embolism and did not reach statistical significance with a HR 0.80 (95% CI 0.61–1.05). In both studies a pre-specified per-protocol analysis was performed, in the STROKESTOP-study a significant reduction in the ischaemic stroke outcome HR 0.76 (95% CI 0.67–0.85) was seen amongst participants compared to control. In the LOOP study, a similar reduction was seen in the group that received the ILR with a HR 0.75 (95% CI 0.56–1.00) compared to controls.

1)  Healthy user bias
  Both studies likely introduced a healthy user bias. In STROKESTOP, it was shown that those who opted to take part in screening were healthier and had a higher socio-economic standing. Also in LOOP participants well enough to attend the screening centre were probably healthier than the population at large.
2)  Detection mode and burden of AF
  In LOOP, the ILR continuously monitored the heart rhythm for more than 3 years. Twice daily rhythm recording in STROKESTOP mounted up to a total of 14 min per individual, i.e. only 0.0008% of the time in LOOP. Accordingly, LOOP was able to reveal AF in 31.8%, compared to only 3% in STROKESTOP. In patients with continuous rhythm monitoring by pacemakers, a lower AF burden has been associated with a more benign outcome.^3^ Therefore, AF episodes detected in STROKESTOP likely identified patients with a large AF burden and thus, a higher risk of stroke. In contrast, many AF episodes detected by the ILR in LOOP were of shorter duration and likely clinically less relevant.
3)  Background detection
  Controls in STROKESTOP were not informed of the intervention ongoing in the screening group. Hence, normal health care-seeking patterns were not disrupted, and AF detection was as expected approximately 1% per year. In the LOOP control group, AF was detected in 12%, a 4-times higher outcome than expected. Owing to the inclusion procedure it is possible that these individuals were especially well informed as to the risks of undiagnosed AF and, therefore, sought after and received similar treatment as the intervention group reducing potential differences between study arms.
4)  Choice of endpoints
  Both studies had a primary outcome including both ischaemic and haemorrhagic strokes and systemic thromboembolism. However, in response to a significant overall trend of decreasing ischaemic strokes in Sweden, the primary endpoint in STROKESTOP was extended to include all-cause mortality and major bleeding. In contrast, the event-driven LOOP study had aimed at a 35% relative risk reduction of the primary endpoint. Although this goal was not met, the difference between the groups was still 20%, an effect size that many would consider a relevant clinical difference. Hence, a type II error cannot be ruled out.

Perhaps less is more—a simplified screening setup using ambulatory easy-to-use digital devices that monitors the heart for shorter periods of time seems able to detect AF that warrants therapy and may be the way forward.

## Figures and Tables

**Figure:**
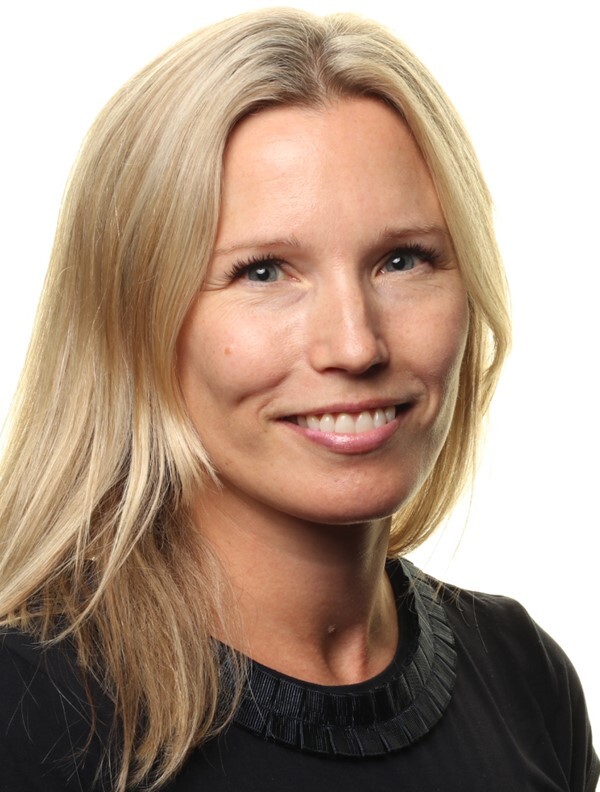


## Lead author biography

Dr Svennberg is currently upholding a combined research and clinical position in the department of electrophysiology at the Karolinska University Hospital in Stockholm, Sweden. Her main clinical and research interests lie within the topic of digital screening for atrial fibrillation. In that capacity she is involved in large screening studies for atrial fibrillation- Strokestop 1 & 2. Dr Svennberg has received the Swedish Society of Cardiology's research award twice for her work. Her interest for detecting asymptomatic atrial fibrillation and improving care for patients with arrhythmias sparked her interest in digital health. She is currently a member of the European Heart Rhythm association's board, and is assigned the role as chair of the Digital Committee. In that capacity she is also liaised to the ESC and Heart Rhythm Society's digital health committees.
